# *N*-(4-Chloro­phen­yl)-4-methyl­benzamide

**DOI:** 10.1107/S1600536811043315

**Published:** 2011-10-29

**Authors:** Vinola Z. Rodrigues, Viktor Vrábel, B. Thimme Gowda, Jozef Kožíšek

**Affiliations:** a Department of Chemistry, Mangalore University, Mangalagangotri 574 199, Mangalore, India; b Institute of Physical Chemistry and Chemical Physics, Slovak University of Technology, Radlinského 9, SK-812 37 Bratislava, Slovak Republic

## Abstract

In the title compound, C_14_H_12_ClNO, the aromatic rings make a dihedral angle of 59.25 (5)°. The methyl group is disordered over two equally occupied positions. In the crystal, N—H⋯O hydrogen bonds link the mol­ecules into infinite *C*(4) chains running along the *a* axis.

## Related literature

For preparation of the title compound, see: Gowda, Jyothi *et al.* (2003 ▶). For studies of the effects of substituents on the structure and other aspects of *N*-(ar­yl)amides, see: Bowes *et al.* (2003 ▶); Gowda *et al.* (2007 ▶); Saeed *et al.* (2010 ▶); of *N*-(ar­yl)methane­sulfonamides, see: Jayalakshmi & Gowda (2004 ▶); of *N*-(ar­yl)aryl­sulfonamides, see: Shetty & Gowda (2005 ▶); and of *N*-chloro­aryl­sulfonamides, see: Gowda, D’Souza & Kumar (2003 ▶).
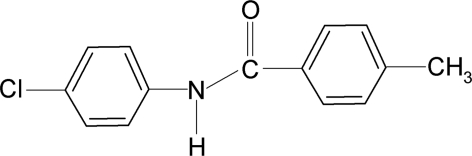


## Experimental

### 

#### Crystal data

C_14_H_12_ClNO
*M* *_r_* = 245.70
Triclinic, 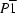

*a* = 5.3837 (3) Å
*b* = 7.7382 (5) Å
*c* = 15.0551 (8) Å
α = 83.146 (5)°
β = 80.436 (4)°
γ = 89.527 (5)°
*V* = 614.03 (6) Å^3^
*Z* = 2
Mo *K*α radiation
μ = 0.29 mm^−1^
*T* = 295 K
0.40 × 0.30 × 0.20 mm

#### Data collection

Oxford Xcalibur diffractometer
Absorption correction: multi-scan (*CrysAlis RED*; Oxford Diffraction, 2009 ▶) *T*_min_ = 0.916, *T*_max_ = 0.952
10437 measured reflections
2506 independent reflections
1947 reflections with *I* > 2σ(*I*)
*R*_int_ = 0.019

#### Refinement

*R*[*F* ^2^ > 2σ(*F* ^2^)] = 0.047
*wR*(*F* ^2^) = 0.152
*S* = 1.08
2506 reflections
159 parameters
1 restraint
H atoms treated by a mixture of independent and constrained refinement
Δρ_max_ = 0.43 e Å^−3^
Δρ_min_ = −0.26 e Å^−3^

### 

Data collection: *CrysAlis CCD* (Oxford Diffraction, 2009 ▶); cell refinement: *CrysAlis CCD*; data reduction: *CrysAlis RED* (Oxford Diffraction, 2009 ▶); program(s) used to solve structure: *SHELXS97* (Sheldrick, 2008 ▶); program(s) used to refine structure: *SHELXL97* (Sheldrick, 2008 ▶); molecular graphics: *DIAMOND* (Brandenburg, 2002 ▶); software used to prepare material for publication: *SHELXL97*, *PLATON* (Spek, 2009 ▶) and *WinGX* (Farrugia, 1999 ▶).

## Supplementary Material

Crystal structure: contains datablock(s) I, global. DOI: 10.1107/S1600536811043315/bt5681sup1.cif

Structure factors: contains datablock(s) I. DOI: 10.1107/S1600536811043315/bt5681Isup2.hkl

Supplementary material file. DOI: 10.1107/S1600536811043315/bt5681Isup3.cml

Additional supplementary materials:  crystallographic information; 3D view; checkCIF report

## Figures and Tables

**Table 1:** Hydrogen-bond geometry (Å, °)

*D*—H⋯*A*	*D*—H	H⋯*A*	*D*⋯*A*	*D*—H⋯*A*
N1—H1⋯O1^i^	0.86 (2)	2.42 (2)	3.184 (2)	148 (2)

Symmetry code: (i) 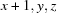.

**Fig. 1.**
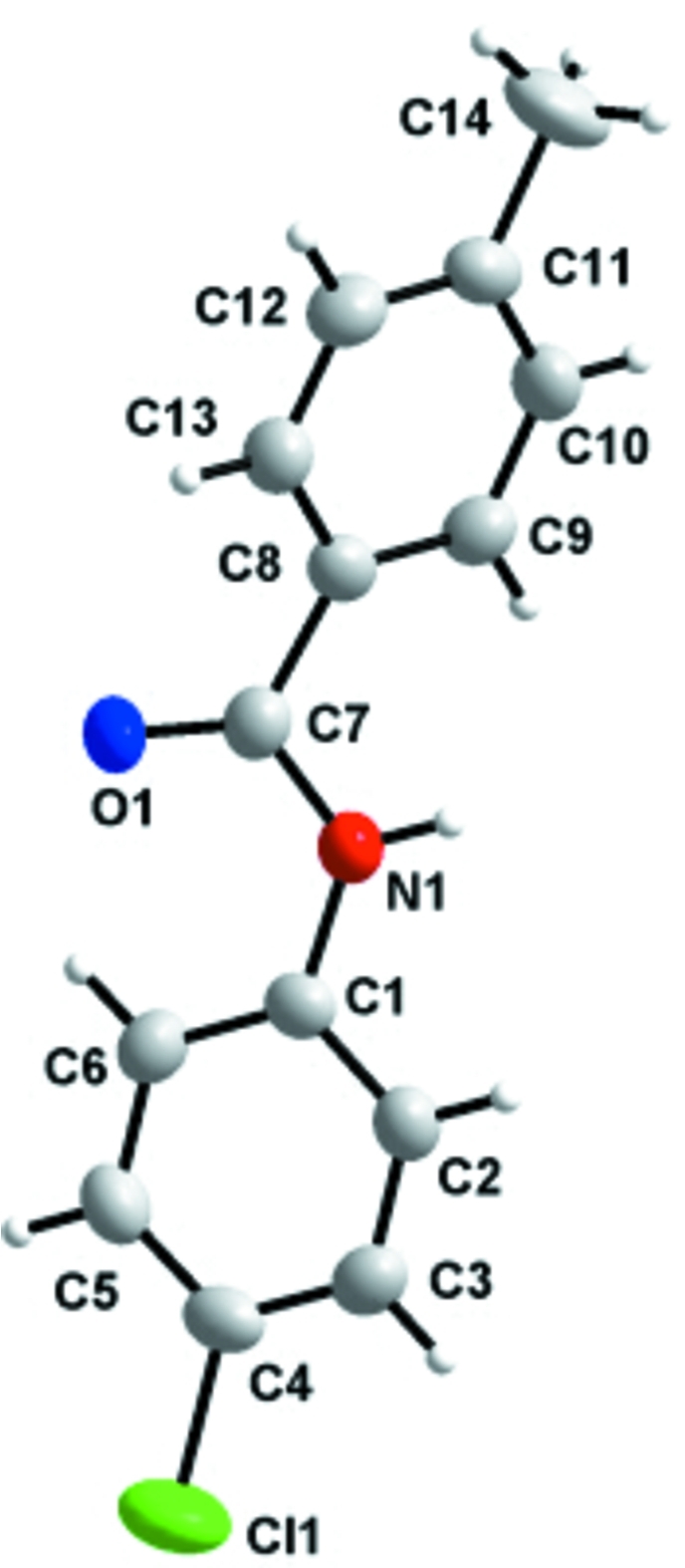
Molecular structure of (I) with the atomic numbering scheme. Displacement ellipsoids are drawn at the 50% probability level. H atoms are represented as small spheres of arbitrary radii.

**Fig. 2.**
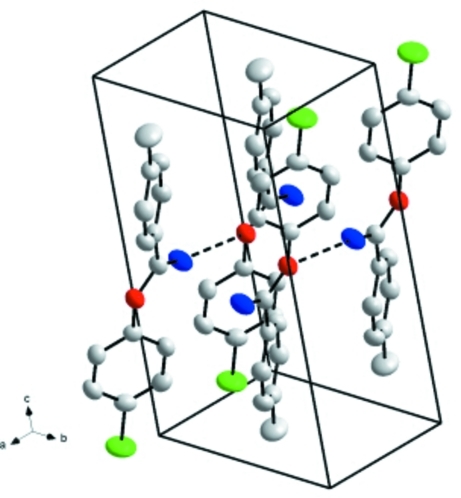
Packing view of the title compound. Molecular chains along a-axis are generated by N—H···O hydrogen bonds which are shown by dashed lines. H atoms have been omitted.

## supplementary crystallographic information

### Comment

The amide and sulfonamide moieties are the constituents of many biologically significant compounds. As part of our studies on the substituent effects on the structures and other aspects of *N*-(aryl)-amides (Bowes *et al.*, 2003; Gowda *et al.*, 2007; Saeed *et al.*, 2010), *N*-(aryl)-methanesulfonamides (Jayalakshmi & Gowda, 2004), *N*-(aryl)-arylsulfonamides (Shetty & Gowda, 2005) and *N*-chloro-arylsulfonamides (Gowda, D'Souza & Kumar, 2003), in the present work, the crystal structure of *N*-(4-Chlorophenyl)-4-methylbenzamide, (I), has been determined (Fig. 1).

In (I), the N—H and C═O bonds are *trans* to each other. The two aromatic rings make a dihedral angle of 59.25 (5)°, while the central amide core –NH—C(═O)– group is twisted by 30.85 (8)° and 28.90 (9)° out of the planes of the 4-chlorphenyl and 4-methyphenyl rings, respectively.

The methyl group is disordered over two equally occupied positions.

In the crystal structure, intermolecular N—H···O hydrogen bonds link the molecules into infinite chains running along the *a*-axis. Part of the crystal structure is shown in Fig. 2.

### Experimental

The title compound was prepared according to the method described by Gowda, Jyothi *et al.* (2003). The purity of the compound was checked by determining its melting point. It was characterized by recording its infrared and NMR spectra. Plate-like colourless single crystals of the title compound were obtained by slow evaporation from an ethanol solution of the compound (0.5 g in about 30 ml of ethanol) at room temperature.

### Refinement

All H atoms except the amide H atom were placed in calculated positions with C—H distances of 0.93 Å (C aromatic), 0.96 Å (C methyl) and constrained to ride on their parent atoms.

The methyl groups of the aromatic ring is disordered over two equally occupied positions rotated with respect to each other by 60°.

The amide H atom was found in a difference map and it was refined isotropically with the N—H distance restrained to 0.86 (2) Å. The *U*_iso_(H) values were set at 1.2*U*_eq_(C aromatic) and 1.5*U*_eq_(C methyl).

### Crystal data

**Table:**

C_14_H_12_ClNO	*Z* = 2
*M**_r_* = 245.70	*F*(000) = 256
Triclinic, *P*1	*D*_x_ = 1.329 Mg m^−^^3^
*a* = 5.3837 (3) Å	Mo *K*α radiation, λ = 0.71073 Å
*b* = 7.7382 (5) Å	Cell parameters from 2506 reflections
*c* = 15.0551 (8) Å	θ = 4.2–26.4°
α = 83.146 (5)°	µ = 0.29 mm^−^^1^
β = 80.436 (4)°	*T* = 295 K
γ = 89.527 (5)°	Plate, colourless
*V* = 614.03 (6) Å^3^	0.40 × 0.30 × 0.20 mm

### Data collection

**Table:**

Oxford Xcalibur diffractometer	2506 independent reflections
Radiation source: fine-focus sealed tube	1947 reflections with *I* > 2σ(*I*)
graphite	*R*_int_ = 0.019
Detector resolution: 0 pixels mm^-1^	θ_max_ = 26.4°, θ_min_ = 4.2°
ω scans with κ offsets	*h* = −6→6
Absorption correction: multi-scan (*CrysAlis RED*; Oxford Diffraction, 2009)	*k* = −9→9
*T*_min_ = 0.916, *T*_max_ = 0.952	*l* = −18→18
10437 measured reflections	

### Refinement

**Table:**

Refinement on *F*^2^	Primary atom site location: structure-invariant direct methods
Least-squares matrix: full	Secondary atom site location: difference Fourier map
*R*[*F*^2^ > 2σ(*F*^2^)] = 0.047	Hydrogen site location: inferred from neighbouring sites
*wR*(*F*^2^) = 0.152	H atoms treated by a mixture of independent and constrained refinement
*S* = 1.08	*w* = 1/[σ^2^(*F*_o_^2^) + (0.0853*P*)^2^ + 0.159*P*] where *P* = (*F*_o_^2^ + 2*F*_c_^2^)/3
2506 reflections	(Δ/σ)_max_ < 0.001
159 parameters	Δρ_max_ = 0.43 e Å^−^^3^
1 restraint	Δρ_min_ = −0.26 e Å^−^^3^

### Special details

**Table:**

Geometry. All e.s.d.'s (except the e.s.d. in the dihedral angle between two l.s. planes) are estimated using the full covariance matrix. The cell e.s.d.'s are taken into account individually in the estimation of e.s.d.'s in distances, angles and torsion angles; correlations between e.s.d.'s in cell parameters are only used when they are defined by crystal symmetry. An approximate (isotropic) treatment of cell e.s.d.'s is used for estimating e.s.d.'s involving l.s. planes.
Refinement. Refinement of *F*^2^ against ALL reflections. The weighted *R*-factor *wR* and goodness of fit *S* are based on *F*^2^, conventional *R*-factors *R* are based on *F*, with *F* set to zero for negative *F*^2^. The threshold expression of *F*^2^ > σ(*F*^2^) is used only for calculating *R*-factors(gt) *etc*. and is not relevant to the choice of reflections for refinement. *R*-factors based on *F*^2^ are statistically about twice as large as those based on *F*, and *R*-factors based on ALL data will be even larger.

### Fractional atomic coordinates and isotropic or equivalent isotropic displacement parameters (Å^2^)

**Table:**

	*x*	*y*	*z*	*U*_iso_*/*U*_eq_	Occ. (<1)
C1	0.3445 (3)	0.2173 (2)	0.37996 (12)	0.0370 (4)	
C2	0.5596 (4)	0.1314 (2)	0.34609 (13)	0.0420 (4)	
H2A	0.6694	0.0887	0.3849	0.050*	
C3	0.6132 (4)	0.1082 (3)	0.25559 (13)	0.0458 (5)	
H3A	0.7579	0.0499	0.2334	0.055*	
C4	0.4512 (4)	0.1719 (3)	0.19831 (13)	0.0446 (5)	
C5	0.2363 (4)	0.2586 (3)	0.23023 (13)	0.0464 (5)	
H5A	0.1284	0.3017	0.1908	0.056*	
C6	0.1820 (4)	0.2812 (3)	0.32122 (13)	0.0436 (5)	
H6A	0.0368	0.3391	0.3431	0.052*	
C7	0.0846 (3)	0.2545 (3)	0.52771 (13)	0.0415 (4)	
C8	0.1024 (3)	0.2816 (2)	0.62364 (12)	0.0383 (4)	
C9	0.3020 (3)	0.3685 (3)	0.64811 (13)	0.0424 (4)	
H9A	0.4370	0.4103	0.6041	0.051*	
C10	0.2992 (4)	0.3926 (3)	0.73789 (14)	0.0457 (5)	
H10A	0.4318	0.4524	0.7534	0.055*	
C11	0.1028 (4)	0.3292 (3)	0.80512 (13)	0.0459 (5)	
C12	−0.0938 (4)	0.2426 (3)	0.77968 (13)	0.0485 (5)	
H12A	−0.2276	0.1990	0.8238	0.058*	
C13	−0.0946 (4)	0.2201 (3)	0.69069 (13)	0.0444 (5)	
H13A	−0.2295	0.1627	0.6753	0.053*	
C14	0.0977 (6)	0.3537 (4)	0.90479 (15)	0.0752 (8)	
H14C	−0.0648	0.3199	0.9390	0.113*	0.50
H14B	0.1301	0.4738	0.9095	0.113*	0.50
H14A	0.2247	0.2829	0.9284	0.113*	0.50
H14F	0.2581	0.3979	0.9123	0.113*	0.50
H14E	0.0632	0.2439	0.9417	0.113*	0.50
H14D	−0.0314	0.4348	0.9229	0.113*	0.50
N1	0.3095 (3)	0.2429 (2)	0.47290 (11)	0.0426 (4)	
H1	0.432 (4)	0.238 (3)	0.5032 (14)	0.051 (6)*	
O1	−0.1177 (2)	0.2432 (2)	0.50164 (10)	0.0556 (4)	
Cl1	0.51597 (14)	0.14345 (10)	0.08371 (4)	0.0811 (3)	

### Atomic displacement parameters (Å^2^)

**Table:**

	*U*^11^	*U*^22^	*U*^33^	*U*^12^	*U*^13^	*U*^23^
C1	0.0389 (9)	0.0370 (9)	0.0357 (9)	−0.0046 (7)	−0.0056 (7)	−0.0076 (7)
C2	0.0402 (10)	0.0432 (10)	0.0454 (10)	0.0028 (8)	−0.0131 (8)	−0.0092 (8)
C3	0.0397 (10)	0.0493 (11)	0.0494 (11)	0.0023 (8)	−0.0034 (8)	−0.0155 (9)
C4	0.0492 (11)	0.0494 (11)	0.0354 (9)	−0.0098 (9)	−0.0034 (8)	−0.0105 (8)
C5	0.0463 (11)	0.0528 (12)	0.0420 (10)	−0.0020 (9)	−0.0157 (8)	−0.0017 (9)
C6	0.0369 (10)	0.0468 (11)	0.0462 (10)	0.0046 (8)	−0.0042 (8)	−0.0066 (8)
C7	0.0354 (9)	0.0445 (11)	0.0454 (10)	0.0001 (8)	−0.0072 (8)	−0.0082 (8)
C8	0.0402 (10)	0.0372 (9)	0.0396 (10)	0.0060 (7)	−0.0094 (8)	−0.0098 (7)
C9	0.0376 (10)	0.0446 (11)	0.0437 (10)	−0.0008 (8)	−0.0009 (8)	−0.0089 (8)
C10	0.0418 (10)	0.0483 (11)	0.0513 (11)	−0.0007 (8)	−0.0133 (8)	−0.0155 (9)
C11	0.0517 (12)	0.0503 (11)	0.0374 (10)	0.0093 (9)	−0.0092 (8)	−0.0103 (8)
C12	0.0440 (11)	0.0551 (12)	0.0435 (11)	−0.0014 (9)	0.0007 (8)	−0.0045 (9)
C13	0.0376 (10)	0.0495 (11)	0.0472 (11)	−0.0031 (8)	−0.0078 (8)	−0.0084 (9)
C14	0.106 (2)	0.0861 (19)	0.0402 (12)	0.0043 (15)	−0.0246 (13)	−0.0180 (12)
N1	0.0349 (8)	0.0531 (10)	0.0422 (9)	0.0023 (7)	−0.0099 (7)	−0.0108 (7)
O1	0.0313 (7)	0.0923 (12)	0.0473 (8)	−0.0016 (7)	−0.0107 (6)	−0.0191 (8)
Cl1	0.0963 (6)	0.1062 (6)	0.0407 (3)	−0.0049 (4)	−0.0022 (3)	−0.0211 (3)

### Geometric parameters (Å, °)

**Table:**

C1—C2	1.384 (3)	C9—C10	1.384 (3)
C1—C6	1.392 (3)	C9—H9A	0.9300
C1—N1	1.418 (2)	C10—C11	1.385 (3)
C2—C3	1.378 (3)	C10—H10A	0.9300
C2—H2A	0.9300	C11—C12	1.387 (3)
C3—C4	1.374 (3)	C11—C14	1.531 (3)
C3—H3A	0.9300	C12—C13	1.372 (3)
C4—C5	1.378 (3)	C12—H12A	0.9300
C4—Cl1	1.7424 (19)	C13—H13A	0.9300
C5—C6	1.384 (3)	C14—H14C	0.9600
C5—H5A	0.9300	C14—H14B	0.9600
C6—H6A	0.9300	C14—H14A	0.9600
C7—O1	1.225 (2)	C14—H14F	0.9600
C7—N1	1.356 (2)	C14—H14E	0.9600
C7—C8	1.503 (2)	C14—H14D	0.9600
C8—C13	1.381 (3)	N1—H1	0.859 (16)
C8—C9	1.394 (3)		
			
C2—C1—C6	119.12 (16)	C12—C11—C14	120.1 (2)
C2—C1—N1	117.24 (16)	C13—C12—C11	121.24 (18)
C6—C1—N1	123.55 (16)	C13—C12—H12A	119.4
C3—C2—C1	120.82 (17)	C11—C12—H12A	119.4
C3—C2—H2A	119.6	C12—C13—C8	120.88 (18)
C1—C2—H2A	119.6	C12—C13—H13A	119.6
C4—C3—C2	119.48 (18)	C8—C13—H13A	119.6
C4—C3—H3A	120.3	C11—C14—H14C	109.5
C2—C3—H3A	120.3	C11—C14—H14B	109.5
C3—C4—C5	120.85 (17)	H14C—C14—H14B	109.5
C3—C4—Cl1	120.06 (16)	C11—C14—H14A	109.5
C5—C4—Cl1	119.10 (15)	H14C—C14—H14A	109.5
C4—C5—C6	119.65 (17)	H14B—C14—H14A	109.5
C4—C5—H5A	120.2	C11—C14—H14F	109.5
C6—C5—H5A	120.2	H14C—C14—H14F	141.1
C5—C6—C1	120.08 (17)	H14B—C14—H14F	56.3
C5—C6—H6A	120.0	H14A—C14—H14F	56.3
C1—C6—H6A	120.0	C11—C14—H14E	109.5
O1—C7—N1	122.97 (18)	H14C—C14—H14E	56.3
O1—C7—C8	122.33 (17)	H14B—C14—H14E	141.1
N1—C7—C8	114.69 (16)	H14A—C14—H14E	56.3
C13—C8—C9	118.61 (17)	H14F—C14—H14E	109.5
C13—C8—C7	117.51 (16)	C11—C14—H14D	109.5
C9—C8—C7	123.86 (17)	H14C—C14—H14D	56.3
C10—C9—C8	120.03 (17)	H14B—C14—H14D	56.3
C10—C9—H9A	120.0	H14A—C14—H14D	141.1
C8—C9—H9A	120.0	H14F—C14—H14D	109.5
C9—C10—C11	121.25 (17)	H14E—C14—H14D	109.5
C9—C10—H10A	119.4	C7—N1—C1	125.82 (16)
C11—C10—H10A	119.4	C7—N1—H1	111.3 (15)
C10—C11—C12	117.98 (17)	C1—N1—H1	122.4 (15)
C10—C11—C14	121.9 (2)		
			
C6—C1—C2—C3	−0.3 (3)	C13—C8—C9—C10	−0.4 (3)
N1—C1—C2—C3	−177.04 (17)	C7—C8—C9—C10	177.90 (17)
C1—C2—C3—C4	0.3 (3)	C8—C9—C10—C11	1.1 (3)
C2—C3—C4—C5	0.1 (3)	C9—C10—C11—C12	−0.9 (3)
C2—C3—C4—Cl1	−179.91 (15)	C9—C10—C11—C14	179.6 (2)
C3—C4—C5—C6	−0.3 (3)	C10—C11—C12—C13	0.0 (3)
Cl1—C4—C5—C6	179.64 (15)	C14—C11—C12—C13	179.5 (2)
C4—C5—C6—C1	0.3 (3)	C11—C12—C13—C8	0.7 (3)
C2—C1—C6—C5	0.0 (3)	C9—C8—C13—C12	−0.5 (3)
N1—C1—C6—C5	176.55 (17)	C7—C8—C13—C12	−178.89 (17)
O1—C7—C8—C13	27.7 (3)	O1—C7—N1—C1	0.2 (3)
N1—C7—C8—C13	−151.95 (18)	C8—C7—N1—C1	179.91 (16)
O1—C7—C8—C9	−150.6 (2)	C2—C1—N1—C7	−150.90 (19)
N1—C7—C8—C9	29.7 (3)	C6—C1—N1—C7	32.5 (3)

### Hydrogen-bond geometry (Å, °)

**Table:**

*D*—H···*A*	*D*—H	H···*A*	*D*···*A*	*D*—H···*A*
N1—H1···O1^i^	0.86 (2)	2.42 (2)	3.184 (2)	148 (2)

Symmetry codes: (i) *x*+1, *y*, *z*.
